# Supplementary figures and images for: Ocean warming affected faunal dynamics of benthic invertebrate assemblages across the Toarcian Oceanic Anoxic Event in the Iberian Basin (Spain)
Source: PLoS One. 2020 Dec 9;15(12):e0242331. doi: 10.1371/journal.pone.0242331 (PMC7725388; doi:10.1371/journal.pone.0242331)

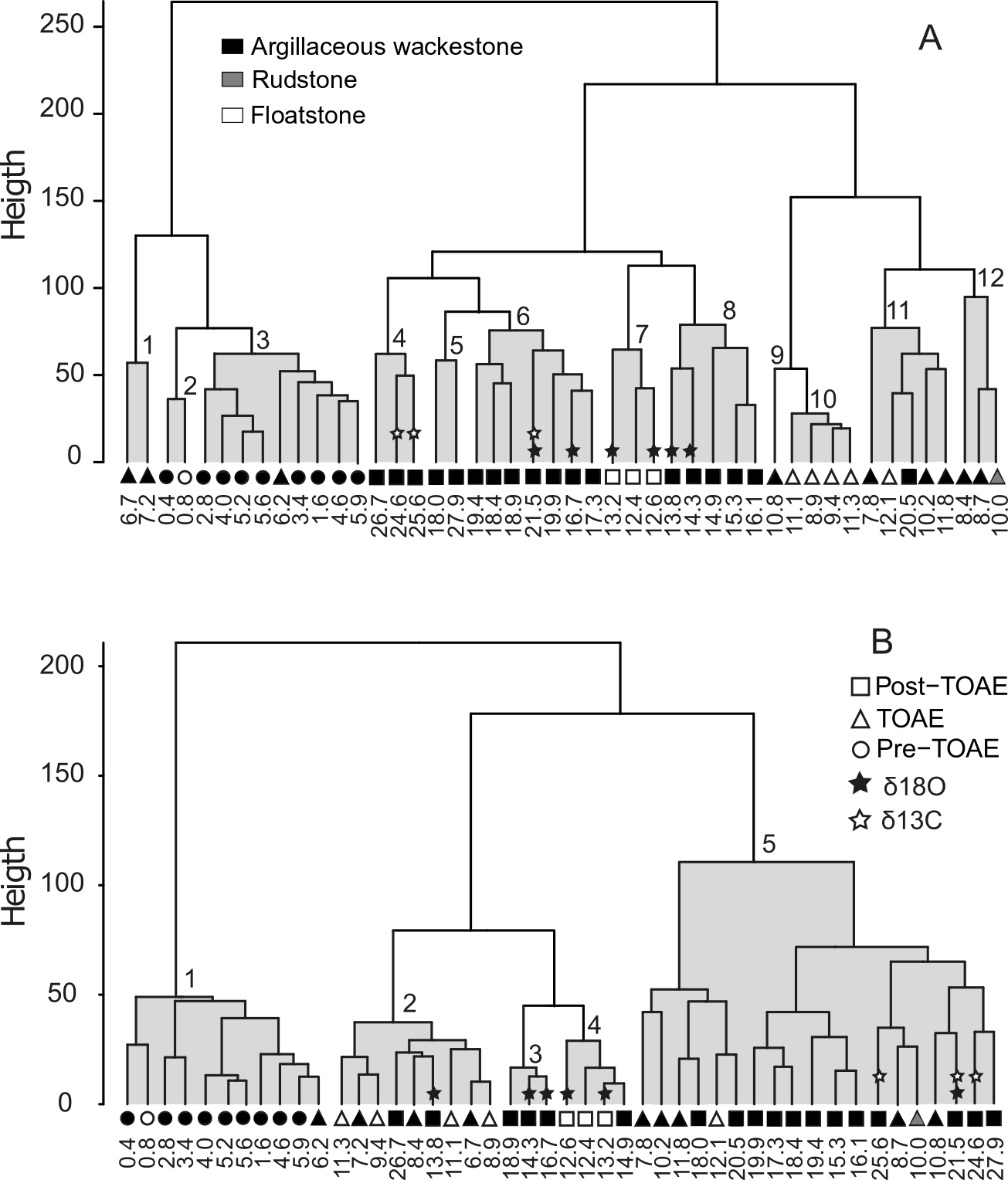

Supplement: S1 Fig — Clusters from Fig 4 plotted showing the stratigraphic level and lithology of each sample in meters above the Pliensbachian-Toarcian boundary. Results plotted by taxonomical (A) and ecological (B) composition. The identified associations are numbered and shaded in grey and each sample is coded by interval (pre-TOAE, TOAE and post-TOAE). The stars mark the levels in the post-TOAE where the recorded isotope values (both δ18O and δ13C) are within pre-TOAE ranges. (TIF) [file pone.0242331.s001.tif]
