# Supplementary material for: Ocean warming affected faunal dynamics of benthic invertebrate assemblages across the Toarcian Oceanic Anoxic Event in the Iberian Basin (Spain)
Source: PLoS One. 2020 Dec 9;15(12):e0242331. doi: 10.1371/journal.pone.0242331 (PMC7725388; doi:10.1371/journal.pone.0242331)
Supplement: S1 Table — Faunal variables are SQS-diversity, evenness, richness, and NMDS axis scores, whereas the geochemical proxy data are δ18O and δ13C values. Results are presented for faunal assemblages characterized by both taxonomic and ecological composition respectively and are based on the original time series. Statistically significant values (p < 0.05) are in bold. (DOCX) [file pone.0242331.s002.docx]

**S1 Table. Results of the Ordinary Least Squares (OLS) correlations of faunal variables and of geochemical proxy data against time (sampling level).**

|  | **Taxonomic composition** | | **Ecological composition** | |
| --- | --- | --- | --- | --- |
|  | ***p*-value** | **Adjusted R^2^** | ***p*-value** | **Adjusted R^2^** |
| SQS ~ Level | **0.013** | 0.1424 | 0.109 | 0.047 |
| Simpson’s Evenness ~ Level | **0.055** | 0.078 | 0.382 | -0.006 |
| Richness ~ Level | 0.942 | -0.029 | 0.408 | -0.009 |
| NMDS1 ~ Level | **<0.001** | 0.768 | **0.028** | 0.109 |
| NMDS2 ~ Level | 0.8231 | -0.028 | **<0.001** | 0.382 |
| δ^18^O ~ Level | 0.4649 | -0.013 | 0.465 | -0.013 |
| δ^13^C ~ Level | **0.005** | 0.188 | **0.005** | 0.188 |
