# Supplementary material for: Ocean warming affected faunal dynamics of benthic invertebrate assemblages across the Toarcian Oceanic Anoxic Event in the Iberian Basin (Spain)
Source: PLoS One. 2020 Dec 9;15(12):e0242331. doi: 10.1371/journal.pone.0242331 (PMC7725388; doi:10.1371/journal.pone.0242331)
Supplement: S2 Table — The OLS correlations are in the form X~ δ18O + δ13C, where X is the respective diversity index or NMDS axis score. Results are presented separately for faunal assemblages characterized by taxonomic and by ecological composition based on the original time series. Statistically significant values (p < 0.05) are in bold. (DOCX) [file pone.0242331.s003.docx]

**S2 Table. Durbin-Watson (D-W) statistics on Ordinary Least Squares (OLS) correlations of faunal variables with geochemical proxy data.**

|  |  | **Taxonomic composition** | | | **Ecological composition** | | |
| --- | --- | --- | --- | --- | --- | --- | --- |
|  | **Lag** | **Autocorrelation** | **D-W Statistic** | ***p*-value** | **Autocorrelation** | **D-W Statistic** | ***p*-value** |
| SQS | 1 | 0.529 | 0.930 | **0.002** | 0.097 | 1.797 | 0.460 |
|  | 2 | 0.460 | 1.060 | **0.004** | -0.020 | 1.943 | 0.884 |
|  | 3 | 0.295 | 1.366 | 0.104 | 0.110 | 1.647 | 0.468 |
|  | 4 | 0.202 | 1.519 | 0.350 | 0.297 | 1.176 | **0.040** |
|  | 5 | 0.083 | 1.673 | 0.740 | 0.022 | 1.634 | 0.676 |
| Simpson’s Evenness | 1 | 0.373 | 1.254 | **0.014** | 0.071 | 1.827 | 0.536 |
|  | 2 | 0.087 | 1.809 | 0.602 | -0.042 | 2.025 | 0.888 |
|  | 3 | 0.006 | 1.957 | 0.880 | -0.076 | 2.090 | 0.550 |
|  | 4 | 0.320 | 1.308 | 0.118 | 0.282 | 1.346 | 0.140 |
|  | 5 | 0.144 | 1.618 | 0.670 | -0.029 | 1.920 | 0.598 |
| Richness | 1 | 0.483 | 1.031 | **0.004** | 0.404 | 1.190 | **0.010** |
|  | 2 | 0.209 | 1.544 | 0.176 | 0.151 | 1.682 | 0.404 |
|  | 3 | 0.041 | 1.824 | 0.780 | -0.048 | 2.071 | 0.672 |
|  | 4 | -0.150 | 2.101 | 0.436 | -0.040 | 1.765 | 0.858 |
|  | 5 | -0.106 | 1.976 | 0.508 | -0.172 | 1.996 | 0.500 |
| NMDS1 | 1 | 0.692 | 0.565 | **0.000** | 0.152 | 1.690 | 0.272 |
|  | 2 | 0.710 | 0.482 | **0.000** | 0.163 | 1.644 | 0.310 |
|  | 3 | 0.514 | 0.771 | **0.000** | 0.245 | 1.449 | 0.186 |
|  | 4 | 0.419 | 0.850 | **0.002** | 0.271 | 1.211 | 0.062 |
|  | 5 | 0.286 | 1.033 | **0.016** | 0.134 | 1.449 | 0.296 |
| NMDS2 | 1 | 0.341 | 1.219 | **0.006** | 0.109 | 1.744 | 0.310 |
|  | 2 | 0.086 | 1.668 | 0.322 | 0.281 | 1.367 | **0.042** |
|  | 3 | -0.008 | 1.799 | 0.710 | -0.101 | 1.953 | 0.980 |
|  | 4 | -0.023 | 1.817 | 0.854 | 0.168 | 1.338 | 0.096 |
|  | 5 | 0.112 | 1.543 | 0.430 | 0.216 | 1.235 | 0.096 |
